# Surgical treatment of cervical rib-associated arterial thoracic outlet syndrome

**DOI:** 10.1590/1677-5449.200106_PT

**Published:** 2021-05-14

**Authors:** Elpidio Ribeiro da Silva, Marcelo Bellini Dalio, Marco Bianco Santarosa, Tércio Ferreira Oliveira, Maurício Serra Ribeiro, Edwaldo Edner Joviliano

**Affiliations:** 1 Universidade de São Paulo – USP, Faculdade de Medicina de Ribeirão Preto, Hospital das Clínicas, Divisão de Cirurgia Vascular e Endovascular, Ribeirão Preto, SP, Brasil.

**Keywords:** thoracic outlet syndrome, subclavian artery, surgery, decompression, cervical rib, emboli, aneurysm

## Abstract

The arterial form of thoracic outlet syndrome is rare and is associated with anatomic anomalies, generally a cervical rib. It has a varied range of manifestations. The aim of this article is to describe two cases with different clinical presentations: microembolization and aneurysm. A cervical rib was present in both cases. Diagnosis was made on the basis of history, physical examination, postural maneuvers, and X-rays. Computed tomography angiography provided the anatomic detail necessary to plan surgery. Surgical treatment was performed via supraclavicular access, successfully, in both cases.

## INTRODUCTION

The arterial form of thoracic outlet syndrome (aTOS) is characterized by compression of the subclavian artery as it passes the scalene triangle and is generally associated with an anatomic anomaly.^1^ It occurs in approximately 1% of cases of the syndrome. Manifestations are highly varied, including stenosis, thrombosis, microembolization, and aneurysm. Treatments for aTOS also vary greatly and depend on the type of clinical presentation.^2^ This article reports on two cases of aTOS with different presentations, both treated successfully. The Research Ethics Committee approved this study (decision number 4.658.058). Both patients gave their consent for publication of their cases.

## DESCRIPTION OF THE CASES

### Case 1

**Figure 1:**
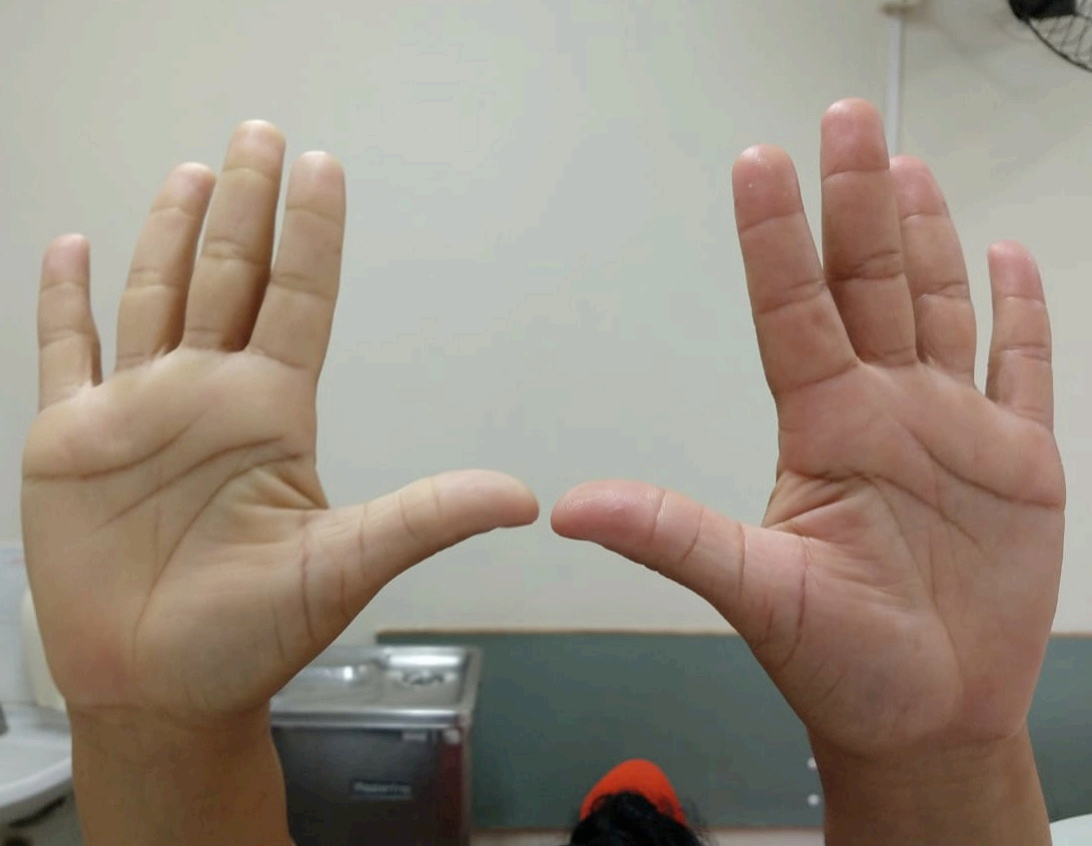
Image showing the hyperabduction maneuver in the first case. The patient raises her arms at 180º. Pallor is observed and the distal pulses disappears in the affected limb (right).

**Figure 2:**
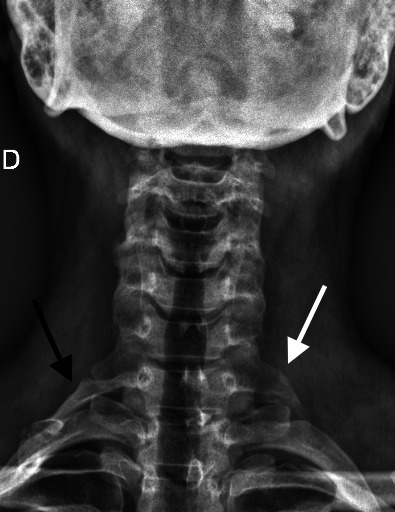
Anteroposterior cervical X-ray of the first case showing the cervical rib joined to the first right rib (black arrow). An elongated transverse process of C7 was also observed on the left (white arrow).

**Figure 3:**
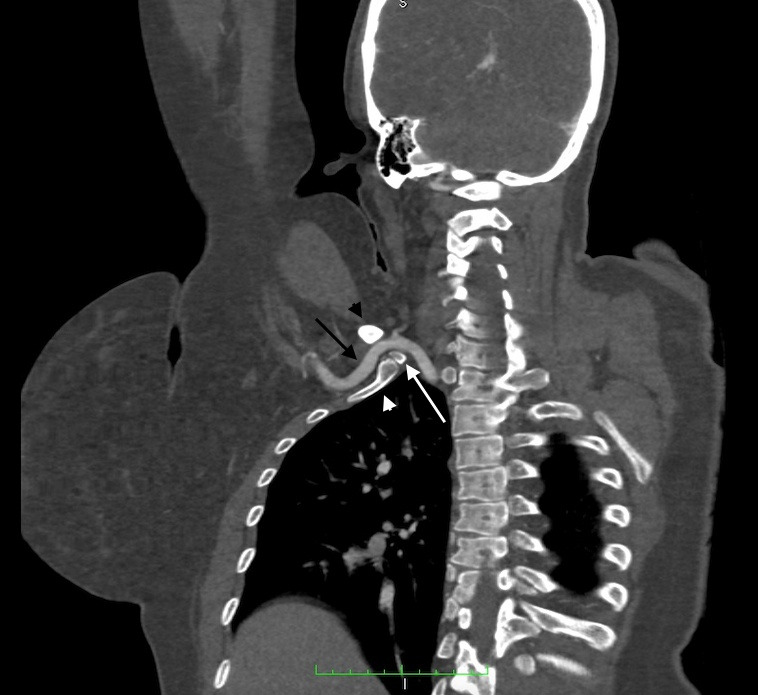
Computed tomography angiography of the thoracic region with upper limb hyperabduction in the first case. The coronal view shows compression of the right subclavian artery (black arrow), by the clavicle (black arrowhead), first rib (white arrowhead), and cervical rib (white arrow). Mild thickening of the artery wall was also observed, but without causing significant stenosis. No aneurysm was observed.

The patient was a 41-year-old female administrative assistant with a history of coldness and pain in the right upper limb with onset 1 year previously and progressive deterioration. Complaints worsened with movement of the upper limb, primarily abduction, limiting the patient’s activities. She reported no comorbidities, local trauma, or smoking. On physical examination, the right hand was pale and cold and capillary refill time was elevated. The right brachial pulse was weaker than its contralateral counterpart and right radial and ulnar pulses were absent, with biphasic flow on duplex imaging. The brachial-brachial index was 0.64. The contralateral upper limb had strong pulses. There was no murmur in the right supraclavicular region. Adson, hyperabduction (Figure 1), and costoclavicular postural maneuvers were positive. The Ross maneuver was negative. There were no neurological changes or muscular atrophy. X-rays of the chest and cervical spine showed a cervical rib joined to the first rib on the right (Figure 2). Computed tomography angiography of the chest with hyperabduction of the upper limbs showed compression of the right subclavian artery by the cervical rib and thickening of the artery wall, without significant stenosis. There was no aneurysm (Figure 3). Having diagnosed aTOS, surgical decompression was indicated.

**Figure 4:**
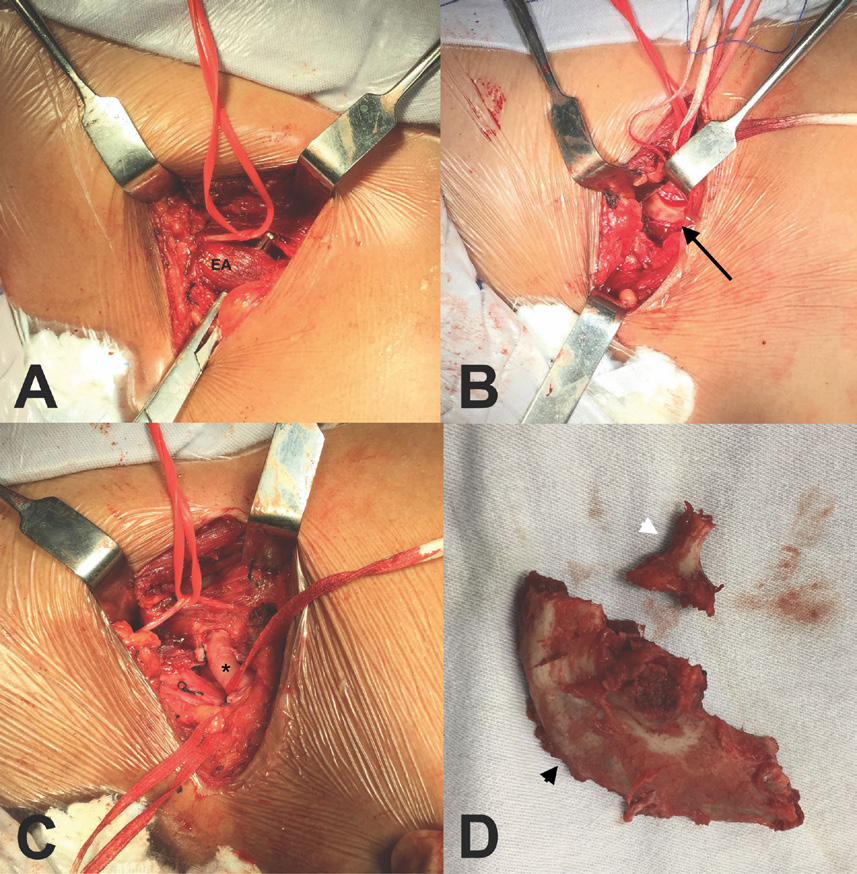
Intraoperative image from case 1. (A) Right supraclavicular approach with observation of the phrenic nerve (red strap) and the anterior scalene muscle (EA); (B) After anterior scalenectomy, the cervical rib was identified (black arrow); (C) After medial scalenectomy and resection of the cervical rib and first rib, the subclavian artery (*) was free from compression, with mild thickening of its wall, but without attenuation of pulsation; (D) Surgical specimens: first rib (black arrowhead) and cervical rib (white arrowhead).

Under general anesthesia, a right supraclavicular approach was obtained, with anterior and medial scalenectomy, followed by total resection of the cervical rib and partial resection of the first rib. The subclavian artery wall was slightly thickened, although without reducing pulsation (Figure 4). Immediately after decompression, the patient’s right brachial pulse was strong and unchanged by abduction of the upper limb. Since the structural change to the artery was discrete and without effect on pulsation, we decided not to resect the injured segment. The patient’s recovery was uneventful and she was prescribed analgesics and motor physiotherapy. She was discharged on the fourth postoperative day. At the 30-day follow-up consultation, she reported no limitation of activities. Her capillary refill times and brachial pulses were symmetrical. Postural maneuvers were negative and her brachial-brachial index was 0.91. She had no wound complications and no motor disorders in the right upper limb. She described mild paresthesia in the fingertips, with progressive improvement after motor physiotherapy. An ultrasound scan at 3 months showed good flow through the right subclavian artery.

### Case 2

**Figure 5:**
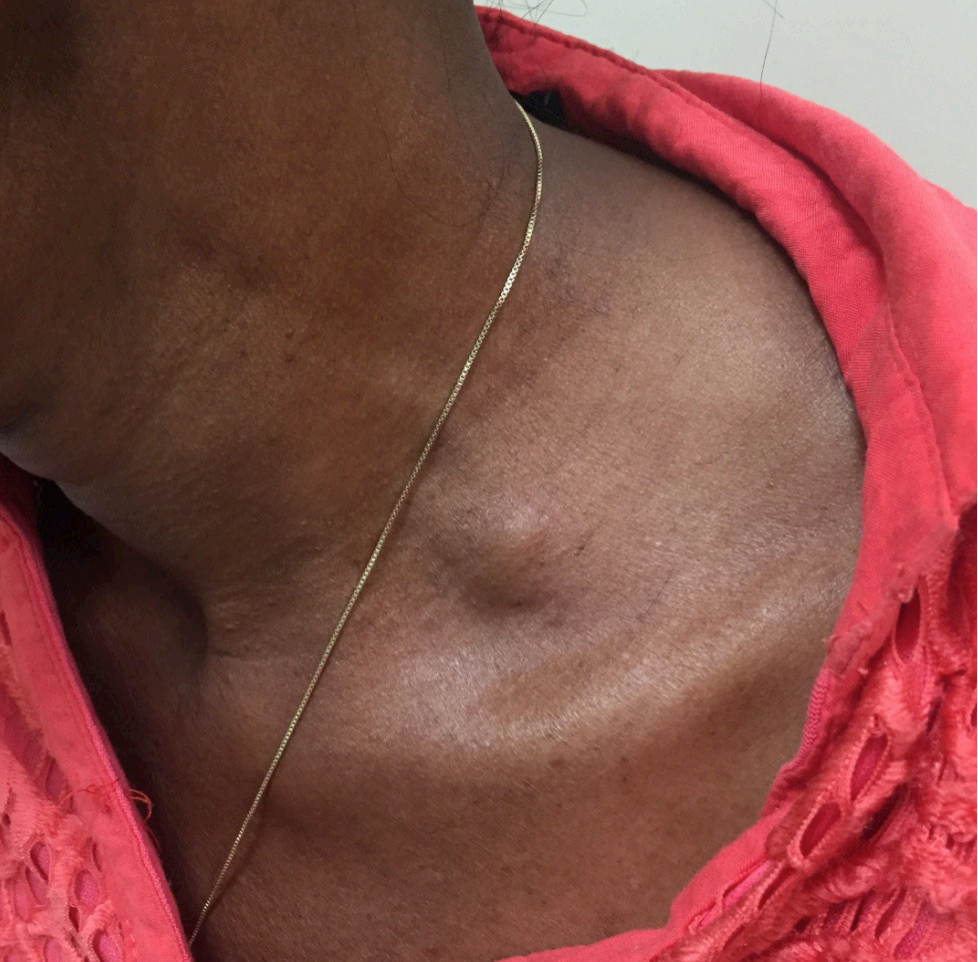
Image of the patient from the second case, showing pulsatile mass in the left supraclavicular fossa.

**Figure 6:**
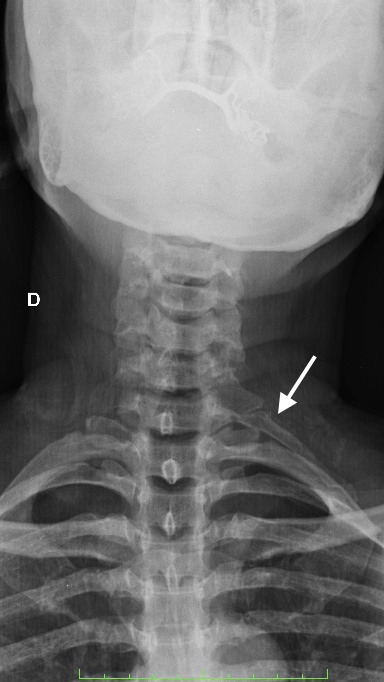
Anteroposterior cervical X-ray from the second case showing a cervical rib joined to the first right rib (white arrow).

**Figure 7:**
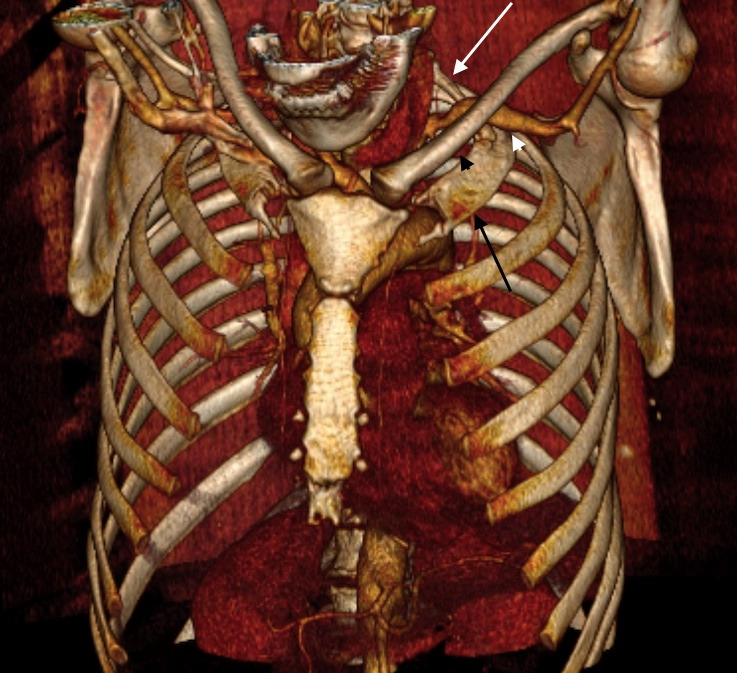
Computed tomography angiography of the thoracic region with upper limbs in hyperabduction, from the second case. The three-dimensional reconstruction shows compression of the left subclavian artery between the clavicle (black arrowhead), first rib (black arrow), and cervical rib (white arrow). Aneurysm formation was observed distal of the compression (white arrowhead).

The patient was a 49-year-old female domestic worker with a 5-year history of a progressively growing mass in the left supraclavicular area. She complained of local pain when moving her left upper limb, worse on cold days. She also complained of paresthesia in the left hand. She reported smoking (140 pack years), but no prior traumas or treatments. Physical examination revealed a pulsatile mass at the left supraclavicular fossa, with an audible murmur on auscultation (Figure 5). Brachial, radial, and ulnar pulses were palpable and symmetrical. The Adson, hyperabduction, costoclavicular, and Roos postural maneuvers were positive for the left upper limb. Perfusion was normal and there was no muscular atrophy and no motor impairment. X-rays of the thorax and cervical spine showed a cervical rib joined to the first left rib (Figure 6). Computed tomography angiography of the chest with hyperabduction of the upper limbs showed the cervical rib compressing the left subclavian artery, which had a distal fusiform aneurysm with a diameter of 2 cm (Figure 7). Surgical treatment was planned with the objective of decompressing the thoracic outlet and repairing the secondary aneurysm.

**Figure 8:**
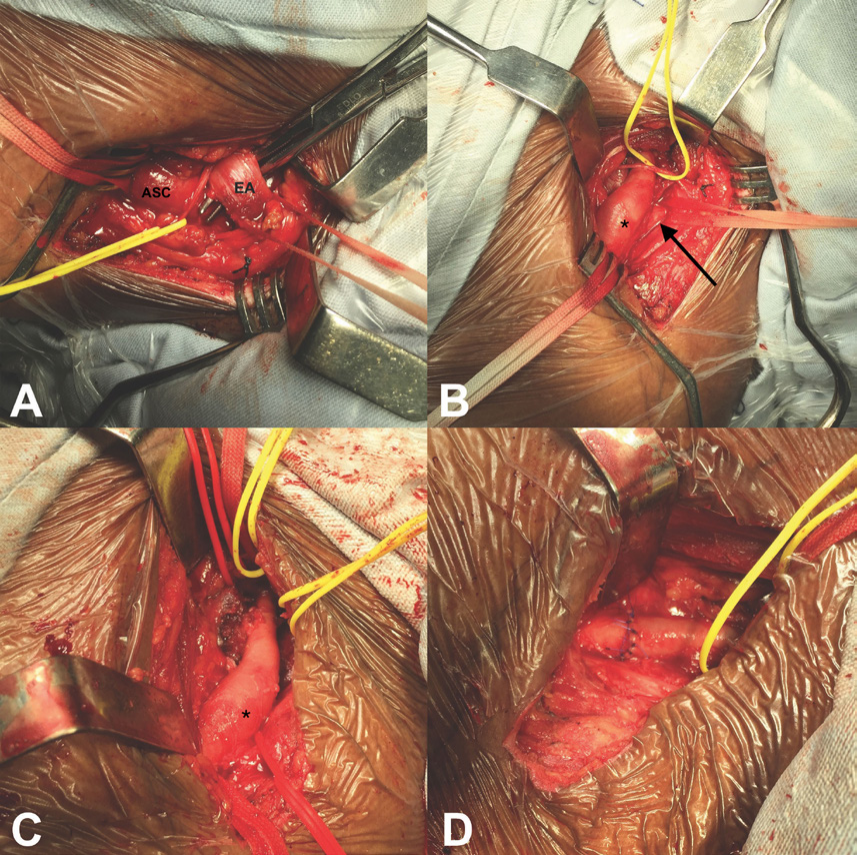
Intraoperative image from case 2. (A) Left supraclavicular approach with observation of the phrenic nerve (yellow strap), the subclavian artery (ASC), and the anterior scalene muscle (EA); (B) After anterior scalenectomy, the cervical rib (black arrow) and aneurysm (*) were identified; (C) After medial scalenectomy and resection of the cervical rib, the aneurysm was identified (*). The outlet was free from compression; (D) The aneurysm was resected and the artery was reconstructed with end-to-end anastomosis with interrupted stitches, using the Carrel triangulation technique.

Under general anesthesia, a left supraclavicular approach was obtained, with anterior and medial scalenectomy, followed by total resection of the cervical rib. After resection of the cervical rib, it was observed that the subclavian artery was free from compression. We therefore decided not to remove the first rib. The aneurysm was treated by resection of the arterial segment involved and reconstruction by end-to-end anastomosis using the Carrel triangulation technique (Figure 8). At the end of the procedure, the patient’s postural maneuvers were negative. The patient was free from postoperative complications and was prescribed analgesics and motor physiotherapy. The patient was discharged on the fourth postoperative day. At the 30-day follow-up consultation, she reported complete absence of the local pain and paresthesia in her left hand. She had no wound complications and pulses were strong and symmetrical. She reported no limitation of activities. An ultrasound scan at 3 months showed good flow through the left subclavian artery.

## DISCUSSION

Compression of the subclavian artery in the scalene triangle is generally associated with an anatomic anomaly, such as a cervical rib, an elongated transverse process of C7, accessory muscle-tendon bundles, and fibrotic bands. Continuous and pulsatile friction of the subclavian artery against bony structures causes fibrosis, stenosis, and aneurysms.^3^ Both cases presented here had cervical ribs joined to the first rib. In the first case, compression caused thickening of the artery wall, and in the second case it caused an aneurysm.

Presentation of aTOS varies greatly.^4^ Patients may develop critical upper limb ischemia and will generally present at emergency. Another form of presentation is distal microembolization. This was the presentation seen in the first case, in which distal pulses were not palpable. Alternatively, patients may develop chronic ischemia of the upper limb in the form of intermittent claudication. Aneurysms can be asymptomatic or may cause localized symptoms, as in the second case. Raynaud’s phenomenon may occur, but this sign is caused by compression of the brachial plexus and is therefore a characteristic of the neurogenic form of the syndrome.^5^

Diagnosis should be made on the basis of history, physical examination, and imaging exams.^6^ Postural maneuvers are useful for diagnosis, but are not pathognomonic. In the cases presented, the maneuvers were positive at presentation and became negative after decompression.

X-rays of the cervical and thoracic regions should be part of initial workup of patients with aTOS. The objective is to detect skeletal anatomic abnormalities. A cervical rib was diagnosed on X-rays in both of the cases presented above.^7^ Duplex ultrasonography has high sensitivity and specificity if conducted in neutral position and with the upper limb in abduction. In addition to changes in flow velocities, it is possible to view artery wall lesions. Computed tomography angiography and magnetic resonance angiography offer precise definition of the position of the injury in the vascular wall and diagnosis of anatomic abnormalities. In common with ultrasonography, these studies should be conducted in neutral position and with abduction. Angiotomography offers superior images of bone structures, while magnetic resonance angiography identifies soft tissues better. It is essential to bear in mind that a finding of positional variation in the caliber of the subclavian artery does not define a diagnosis of aTOS. There must be damage to the artery wall and correlation with clinical presentation. Although arteriography is the gold standard, it is being used less and less to investigate aTOS.^8^ In the cases described here, angiotomography enabled precise diagnosis and treatment planning.

Treatment of aTOS is surgical. It is essential to achieve decompression of the thoracic outlet with correction of anatomic anomalies. Generally, a segment of the first rib is removed to enlarge the space available. When necessary, appropriate arterial reconstruction should be performed during the same operation.^9^ Decompression can be performed via supraclavicular or transaxillary approaches. The supraclavicular approach provides ample exposure of the structures of the thoracic outlet, enabling resection of the anterior and medial scalene muscles, the cervical rib, and the first rib. It also offers sufficient exposure for vascular reconstruction. It has the disadvantage of involving manipulation of the brachial plexus. For these reasons, this was the approach chosen in both cases and is generally the preferred access for aTOS.^9^ The transaxillary approach offers the advantage of decompressing the thoracic outlet by removal of the first rib, without manipulation of neurovascular structures. This procedure has recently been performed with the aid of videothoracoscopy.^10^ Since this approach does not allow arterial reconstruction or resection of a cervical rib, its indications for aTOS are limited. After decompression, the artery wall injury should be assessed. As in the first case described above, thickening and fibrosis of the artery wall can be treated conservatively and monitored with imaging exams. Stenosis and aneurysms can be treated with resection and/or interposition of great saphenous vein or synthetic grafts. In the second case described above, the aneurysm was resected and end-to-end anastomosis performed. Angioplasty with or without stenting is not recommended as primary treatment for aTOS because compression by bone structures leads to stent fracture and thrombosis. The mechanical forces in play at the thoracic outlet are easily capable of fracturing even the most resistant stents.

## CONCLUSIONS

The cases of aTOS described here presented with distal microembolization and aneurysm, both associated with cervical ribs. Diagnosis was based on history, physical examination, and X-rays. Computed tomography angiography provided the level of anatomic detail needed to plan the operations. Surgical treatment via the supraclavicular approach was effective.

## References

[1] Illig KA, Donahue D, Duncan A (2016). Reporting standards of the Society for Vascular Surgery for thoracic outlet syndrome. J Vasc Surg.

[2] Dalio MB, Piccinato CE, Joviliano EE, Moriya T, Ribeiro MS (2019). Síndrome do desfiladeiro torácico.. Manual prático de angiologia e cirurgia vascular..

[3] Freischlag J, Orion K (2014). Understanding thoracic outlet syndrome. Scientifica.

[4] Thomazinho F, Sardinha WE, Silvestre JMS, Morais D, Motta F (2008). Complicações arteriais da síndrome do desfiladeiro torácico. J Vasc Bras.

[5] Vemuri C, McLaughlin LN, Abuirqeba AA, Thompson RW (2017). Clinical presentation and management of arterial thoracic outlet syndrome. J Vasc Surg.

[6] Sanders RJ, Hammond SL, Rao NM (2007). Diagnosis of thoracic outlet syndrome. J Vasc Surg.

[7] Raptis CA, Sridhar S, Thompson RW, Fowler KJ, Bhalla S (2016). Imaging of the patient with thoracic outlet syndrome. Radiographics.

[8] Ghouri MA, Gupta N, Bhat AP (2019). CT and MR imaging of the upper extremity vasculature: pearls, pitfalls, and challenges. Cardiovasc Diagn Ther.

[9] Hussain MA, Aljabri B, Al-Omran M (2016). Vascular thoracic outlet syndrome. Semin Thorac Cardiovasc Surg.

[10] Ghefter MC, Yoshida WB, Cataneo DC (2012). Síndrome do desfiladeiro torácico: ressecção de costela cervical por videotoracoscopia. J Vasc Bras.

