# Supplementary material for: Surgical treatment of cervical rib-associated arterial thoracic outlet syndrome
Source: J Vasc Bras. 2021 May 14;20:e20200106. doi: 10.1590/1677-5449.200106_PT (PMC8147708; doi:10.1590/1677-5449.200106_PT)
Supplement: Translation

# Translation: Tratamento cirúrgico da forma arterial da síndrome do desfiladeiro torácico associado à costela cervical

**DOI:** 10.1590/1677-5449.200106_EN

**Authors:** Elpidio Ribeiro da Silva, Marcelo Bellini Dalio, Marco Bianco Santarosa, Tércio Ferreira Oliveira, Maurício Serra Ribeiro, Edwaldo Edner Joviliano

**Affiliations:** 1 Universidade de São Paulo – USP, Faculdade de Medicina de Ribeirão Preto, Hospital das Clínicas, Divisão de Cirurgia Vascular e Endovascular, Ribeirão Preto, SP, Brasil.

**Keywords:** síndrome do desfiladeiro torácico, artéria subclávia, cirurgia, descompressão, costela cervical, embolia, aneurisma

## Abstract

A forma arterial da síndrome do desfiladeiro torácico é rara e está associada a uma anomalia anatômica, geralmente uma costela cervical. Suas manifestações são muito variadas. Este artigo tem como proposta relatar dois casos de apresentações clínicas distintas: microembolização e aneurisma. Em ambos, uma costela cervical estava presente. O diagnóstico foi realizado através da história, do exame físico, das manobras posturais e das radiografias. A angiotomografia computadorizada proporcionou o detalhe anatômico necessário para o planejamento operatório. O tratamento cirúrgico foi realizado pela abordagem supraclavicular, com sucesso em ambos casos.

## INTRODUÇÃO

A forma arterial da síndrome do desfiladeiro torácico (aSDT) é caracterizada pela compressão da artéria subclávia ao passar pelo trígono interescalênico e geralmente está associada a uma anomalia anatômica^1^. Ocorre em aproximadamente 1% dos casos dessa síndrome. Suas manifestações são muito variadas, abrangendo estenose, trombose, microembolização e aneurisma. O tratamento da aSDT também varia muito e depende de cada tipo de apresentação clínica^2^. Este artigo tem como proposta relatar dois casos de apresentações distintas de aSDT tratados com sucesso. O protocolo foi aprovado pelo Comitê de Ética de nossa instituição (parecer nº 4.658.058). Os pacientes consentiram com a publicação dos casos.

## DESCRIÇÃO DOS CASOS

### Caso 1

Paciente do sexo feminino, 41 anos, auxiliar administrativa, apresentava história de um ano de frialdade e dor no membro superior (MS) direito, com piora progressiva. As queixas pioravam com a movimentação do MS, principalmente à abdução, limitando as suas atividades. Negava comorbidades, trauma local ou tabagismo. Ao exame físico, apresentava palidez, frialdade e aumento do tempo de enchimento capilar na mão direita. O pulso braquial direito era reduzido em relação ao contralateral, e os pulsos radial e ulnar direitos eram ausentes, com fluxo bifásico no Doppler. O índice bráquio-braquial era 0,64. O MS contralateral tinha os pulsos amplos. Não havia sopro na região supraclavicular direita. As manobras posturais de Adson, hiperabdução (Figura 1) e costoclavicular foram positivas. A manobra de Ross foi negativa. Não havia alterações neurológicas ou atrofia muscular. As radiografias de tórax e de coluna cervical evidenciaram costela cervical articulada com a primeira costela à direita (Figura 2). A angiotomografia computadorizada do tórax com hiperabdução dos membros superiores (MMSS) mostrou compressão da artéria subclávia direita pela costela cervical e espessamento da parede arterial sem determinar estenose significativa. Não havia aneurisma (Figura 3). Com o diagnóstico de aSDT, foi indicada a descompressão cirúrgica.

**Figura 1:** Imagem da manobra de hiperabdução do primeiro caso. A paciente eleva os membros superiores em 180º. Observa-se palidez e desaparecimento dos pulsos distais no membro afetado (direito).

**Figura 2:** Radiografia cervical anteroposterior do primeiro caso mostrando costela cervical articulada com primeira costela à direita (seta preta). Observou-se também processo transverso alongado de C7 à esquerda (seta branca).

**Figura 3:** Angiotomografia computadorizada da região torácica com hiperabdução dos membros superiores do primeiro caso. O corte coronal mostrou compressão da artéria subclávia direita (seta preta), pela clavícula (cabeça de seta preta), primeira costela (cabeça de seta branca) e costela cervical (seta branca). Observou-se leve espessamento da parede arterial sem determinar estenose significativa. Não foi observado aneurisma.

Sob anestesia geral, foram realizados acesso supraclavicular direito, escalenectomia anterior e média, ressecção total da costela cervical e parcial da primeira costela. Foi identificado leve espessamento da parede da artéria subclávia sem determinar redução de sua pulsação (Figura 4). Imediatamente após a descompressão, a paciente apresentava pulso braquial direito amplo sem alteração à abdução do MS. Como a alteração estrutural na artéria era discreta e sem repercussão na pulsação, optamos por não ressecar o segmento lesado. A paciente apresentou evolução sem intercorrências, recebendo analgesia e fisioterapia motora. Recebeu alta no quarto dia pós-operatório. No retorno, 30 dias após, a paciente negou limitação às atividades. O tempo de enchimento capilar e os pulsos braquiais eram simétricos. As manobras posturais foram negativas e o índice bráquio-braquial era 0,91. A ferida não apresentava complicações e não havia alterações motoras no MS direito. Relatou leve parestesia na ponta dos quirodáctilos, com melhora progressiva após a fisioterapia motora. O ultrassom realizado após três meses mostrou fluxo adequado na artéria subclávia direita.

**Figura 4:** Imagem intraoperatória do caso 1. (A) Abordagem supraclavicular direita com reparo do nervo frênico (alça vermelha) e do músculo escaleno anterior (EA); (B) Após a escalenectomia anterior, identificou-se a costela cervical (seta preta); (C) Após a escalenectomia média e a ressecção da costela cervical e da primeira costela, a artéria subclávia (*) estava livre de compressão, havendo leve espessamento de sua parede sem determinar redução de sua pulsação; (D) Peça cirúrgica: primeira costela (cabeça de seta preta) e costela cervical (cabeça de seta branca).

### Caso 2

Paciente do sexo feminino, 49 anos, doméstica, apresentava história de 5 anos de abaulamento na região supraclavicular esquerda, com aumento progressivo. Referia dor local durante a movimentação do MS esquerdo com piora nos dias frios. Queixava-se também de parestesia na mão esquerda. Referia tabagismo (140 anos-maço). Negava traumas ou tratamentos prévios. Ao exame físico, apresentava abaulamento pulsátil na fossa supraclavicular esquerda, com sopro à ausculta (Figura 5). Os pulsos braquiais, radiais e ulnares eram palpáveis e simétricos. As manobras posturais de Adson, hiperabdução, costoclavicular e Roos eram positivas no MS esquerdo. A perfusão era normal, e não havia atrofia muscular ou alterações motoras. Foram realizadas radiografias de tórax e coluna cervical, as quais evidenciaram costela cervical articulada com primeira costela à esquerda (Figura 6). A angiotomografia computadorizada do tórax com manobra de hiperabdução dos MMSS mostrou a costela cervical comprimindo a artéria subclávia esquerda com aneurisma fusiforme distal com diâmetro de 2 cm (Figura 7). Foi indicado o tratamento cirúrgico com o objetivo de descomprimir o desfiladeiro torácico e corrigir o aneurisma secundário.

**Figura 5:** Imagem da paciente do segundo caso mostrando abaulamento pulsátil na fossa supraclavicular esquerda.

**Figura 6:** Radiografia cervical anteroposterior do segundo caso mostrando costela cervical articulada com primeira costela à esquerda (seta branca).

**Figura 7:** Angiotomografia computadorizada da região torácica com hiperabdução dos membros superiores do segundo caso. A reconstrução tridimensional mostrou compressão da artéria subclávia esquerda entre clavícula (cabeça de seta preta), primeira costela (seta preta) e costela cervical (seta branca). Observou-se a formação de aneurisma distal à compressão (cabeça de seta branca).

Sob anestesia geral e pelo acesso supraclavicular esquerdo, a paciente foi submetida a escalenectomia anterior e média e ressecção total da costela cervical. Após a ressecção da costela cervical, observou-se que a artéria subclávia estava livre de compressão. Optamos, então, por não remover a primeira costela. O aneurisma foi tratado pela ressecção do segmento arterial acometido e reconstrução por anastomose término-terminal por triangulação de Carrel (Figura 8). Ao fim do procedimento, a paciente apresentava manobras posturais negativas. A paciente não apresentou intercorrências pós-operatórias, recebeu analgesia e fisioterapia motora. A paciente foi liberada para casa no quarto dia pós-operatório. No retorno, 30 dias após o procedimento, referiu melhora total da dor local e da parestesia na mão esquerda. A ferida não apresentava complicações, e os pulsos eram amplos e simétricos. Negava limitação às atividades. O ultrassom realizado após três meses mostrou fluxo adequado na artéria subclávia esquerda.

**Figura 8:** Imagem intraoperatória do caso 2. (A) Abordagem supraclavicular esquerda com reparo do nervo frênico (alça amarela), da artéria subclávia (ASC) e do músculo escaleno anterior (EA); (B) Após a escalenectomia anterior, identificou-se a costela cervical (seta preta) e o aneurisma (*); (C) Após a escalenectomia média e a ressecção da costela cervical, identificou-se o aneurisma (*). O desfiladeiro estava livre de compressão; (D) O aneurisma foi ressecado e a artéria foi reconstruída com anastomose término-terminal com pontos separados pela técnica da triangulação de Carrel.

## DISCUSSÃO

A compressão da artéria subclávia no triângulo interescalênico é geralmente associada a uma anomalia anatômica, como a costela cervical, o processo transverso alongado de C7, os feixes músculo-tendíneos acessórios e as bandas fibróticas. A fricção contínua e pulsátil da artéria subclávia com as estruturas ósseas causa fibrose, estenose e aneurisma^3^. Ambos os casos apresentados tinham costela cervical, articulada com a primeira costela. No primeiro, a compressão causou espessamento da parede arterial, e no segundo, causou um aneurisma.

A apresentação da aSDT é muito variada^4^. Os pacientes podem desenvolver isquemia crítica do MS e geralmente procuram a emergência. Outra forma de apresentação é a microembolização distal. Essa foi a apresentação do primeiro caso, que não tinha pulsos distais palpáveis. Alternativamente, os pacientes podem desenvolver isquemia crônica do MS na forma de claudicação intermitente. Os aneurismas podem ser assintomáticos ou causar sintomas locais, como no segundo caso. O fenômeno de Raynaud pode ocorrer, mas esse sinal se deve à compressão do plexo braquial, sendo, assim, uma característica da forma neurogênica da síndrome^5^.

O diagnóstico deve ser baseado na história, no exame físico e nos exames de imagem^6^. As manobras posturais são úteis para o diagnóstico; entretanto, não são patognomônicas. Nos casos apresentados, as manobras eram positivas e tornaram-se negativas após a descompressão.

As radiografias da região cervical e torácica devem fazer parte da avaliação inicial dos pacientes com aSDT. O objetivo é detectar alterações anatômicas ósseas. Em ambos os casos apresentados, a costela cervical foi diagnosticada pela radiografia^7^. A ultrassonografia com Doppler, realizada nas posições neutra e em abdução do MS, tem alta sensibilidade e especificidade. Além da alteração nas velocidades de fluxo, é possível visualizar a lesão na parede arterial. A angiotomografia computadorizada e a angiorressonância magnética possibilitam a definição precisa da lesão na parede vascular e o diagnóstico das alterações anatômicas. Da mesma maneira que a ultrassonografia, esses estudos devem ser realizados em posição neutra e em abdução. A angiotomografia proporciona imagens superiores das estruturas ósseas, enquanto a angiorressonância identifica melhor as partes moles. É fundamental lembrar que o achado de variação posicional do calibre da artéria subclávia não define o diagnóstico da aSDT. É necessário haver lesão na parede arterial e correlação com a apresentação clínica. Apesar de ser o padrão-ouro, a arteriografia é cada vez menos utilizada na avaliação da aSDT^8^. Nos casos descritos, a angiotomografia possibilitou o diagnóstico preciso e o planejamento terapêutico.

O tratamento da aSDT é cirúrgico. É fundamental realizar a descompressão do desfiladeiro torácico com a correção das anomalias anatômicas. Geralmente, um segmento da primeira costela é removido no intuito de ampliar o espaço. A reconstrução arterial apropriada, quando necessária, deve ser realizada no mesmo ato^9^. A descompressão pode ser realizada pelas abordagens supraclavicular ou transaxilar. A abordagem supraclaviclar proporciona uma exposição ampla das estruturas do desfiladeiro torácico, o que possibilita a ressecção dos músculos escaleno anterior e médio, da costela cervical e da primeira costela. Permite também a exposição adequada para reconstrução vascular. Tem a desvantagem da manipulação do plexo braquial. Por essas razões, essa foi a abordagem escolhida para os casos descritos e é geralmente a preferida na aSDT^9^. A abordagem transaxilar tem a vantagem de descomprimir o desfiladeiro torácico por meio da remoção da primeira costela, sem a manipulação das estruturas neurovasculares. Recentemente, esse procedimento tem sido realizado com auxílio da videotoracoscopia^10^. Como essa abordagem não possibilita a reconstrução arterial nem a ressecção da costela cervical, ela tem indicações restritas na aSDT. Após a descompressão, a lesão na parede arterial deve ser avaliada. Como no primeiro caso, o espessamento e a fibrose da parede arterial podem ser tratados de maneira conservadora e acompanhamento com exames de imagem. Estenoses e aneurismas podem ser tratados com ressecção e/ou interposição de enxerto de veia safena magna ou sintético. No segundo caso, foi realizada a resseção do aneurisma e anastomose término-terminal. A angioplastia com ou sem colocação de stents é desaconselhada como tratamento primário da aSDT. A compressão por estruturas ósseas leva à fratura e à trombose do stent. As forças mecânicas do desfiladeiro torácico são capazes de fraturar facilmente até o stent mais resistente.

## CONCLUSÃO

Os casos descritos da aSDT tiveram como apresentação microembolização distal e aneurisma, ambos associados à costela cervical. O diagnóstico foi baseado história, no exame físico e nas radiografias. A angiotomografia computadorizada proporcionou o detalhe anatômico necessário para o planejamento operatório. O tratamento cirúrgico pela abordagem suplaclavicular foi efetivo.
